# Validation of the Turkish version of the Liverpool Oral Rehabilitation Questionnaire version 3 (LORQv3) in prosthetically rehabilitated patients with head and neck cancer

**DOI:** 10.1186/1472-6831-14-129

**Published:** 2014-10-22

**Authors:** Kadriye Peker, Meltem Ozdemir-Karatas, Ali Balık, Esma Kürklü, Ömer Uysal, Simon N Rogers

**Affiliations:** Department of Dental Public Health, Faculty of Dentistry, Istanbul University, Çapa, Istanbul, Turkey; Department of Maxillofacial Prosthodontics, Faculty of Dentistry, Istanbul University, Çapa, Istanbul, Turkey; Department of Oral Surgery, Faculty of Dentistry, Istanbul University, Çapa, Istanbul, Turkey; Department of Medical Statistics and Informatics, Medical School, Bezmialem Vakif University, Fatih, Istanbul, Turkey; Regional Head and Neck Unit, University Hospital Aintree, Longmoor Lane, Liverpool, L9 7AL UK; Evidence-Based Practice Research Centre (EPRC), Edge Hill University, Lancashire, UK

**Keywords:** Quality of life, Oral rehabilitation, Cultural adaptation, Reliability, Validity, Patients with head and neck cancer

## Abstract

**Background:**

The Liverpool Oral Rehabilitation Questionnaire version 3 (LORQv3) is a measure assessing the impact of oral rehabilitation on patients’ health-related quality of life (HRQOL).The aims of the study were to adapt culturally the LORQv3 for Turkish-speaking head and neck cancer patients who had undergone prosthetic rehabilitation and to undertake an initial investigation of its psychometric properties.

**Methods:**

The Turkish version of the LORQv3 was translated and culturally adapted into Turkish, and tested on a sample of 46 head and neck cancer patients who had undergone prosthetic rehabilitation at a university clinic. Patients were categorized into three groups: Patients with maxillary obturator prostheses treated by surgery alone (n = 15); Patients with maxillary obturator prostheses treated by surgery plus radiotherapy, with or without chemotherapy (n = 23); and, Nasopharyngeal cancer patients without maxillary defects wearing conventional dental prostheses who had been treated by radiotherapy with or without chemotherapy (n = 8). Data were collected through clinical examinations and self-reported questionnaires, including socio-demographic characteristics, the LORQv3, and the University of Washington Quality of Life questionnaire version 4 (UW-QOLv4). The psychometric evaluation included validity (content, face, construct, and criterion) and reliability (internal consistency and test-retest).

**Results:**

All sections of the LORQv3 showed satisfactory internal consistency, with Cronbach’s alpha between 0.71 to 0.82. Kappa statistics showed moderate to perfect test-retest reliability for the 33 LORQv3 items. We found significant negative correlations between the LORQv3 and the UW-QOL v4 for some related items. The LORQv3 also identified differences in responses among patient groups, supporting its construct and criterion validity.

**Conclusions:**

This study provides initial evidence in support of the validity and reliability of the Turkish version of LORQv3 in prosthetically rehabilitated patients with head and neck cancer; it could be used in clinical practice in Turkey.

## Background

In recent years, there has been increasing recognition that health-related quality of life measures (HRQOL) play an important role for assessing clinically significant changes in cancer patients and the comparative effectiveness of different treatments [1, 2]. Following treatment, patient concerns shift from survival towards improvement and maintenance of the HRQOL [3]. In this period, oral rehabilitation is a cornerstone of efforts to restore patients’ orofacial form and function, assisting them to face functional, psychosocial and aesthetic problems that affect general well-being [1, 3, 4].

Generic HRQOL measures are commonly used in a combination with head and neck cancer and oral health specific HRQOL measures in many studies assessing treatment outcomes of oral rehabilitation on patients’ HRQOL [5–11]. A recent consensus report on orofacial rehabilitation [12] states that more sensitive and specific measures are needed for assessing the impacts of oral rehabilitation on patients’ HRQOL, because the existing measures seem to lack discriminating ability to measure the effects of oral rehabilitation on HRQOL in these cases. In this context, the Liverpool Oral Rehabilitation Questionnaire (LORQ) is a recently developed HRQOL measure that deal specifically with oral rehabilitation for patients with head and neck cancer [13, 14]. This measure was designed to identify issues and problems pertaining to the oral rehabilitation of patients within the context of the patients’ overall HRQOL [14]. Since its publication in 2004, the questionnaire has been modified to include more detailed questions on oral function and patients’ dental and prosthetic status, resulting in the LORQ version 3 (LORQ v3) [15]. The LORQv3 has been validated for both dental patients attending general dental practices and patients attending the oral rehabilitation clinic [15–17].

In Turkey, demand for cancer treatment has been rising because of the increasing trends in cancer rates [18]. To our knowledge, there are no published studies that have evaluated the impacts of oral rehabilitation on head and neck cancer patients’ HRQOL using head and neck cancer specific measures in Turkey. The aims of this study were to carry out the cultural adaptation of the LORQv3 into Turkish and to assess its reliability and validity in patients with head and neck cancer who underwent oral rehabilitation.

## Methods

The study was performed in two stages. In the first stage, the scale was translated into Turkish and adapted to Turkish culture. In the second stage, the psychometric properties of the LORQv3 was evaluated.

### Translation and adaptation process

We followed the six steps described by Beaton et al. [19], intended for questionnaires of self-report health measures, as follows:

Stage 1- Two independent native Turkish speaking translators were used to translate the LORQv3 into Turkish. One of the translators was a clinician and therefore aware of the concepts that are being measured with the LORQv3 and the other translator was a language specialist without medical background.

Stage 2- The two translators met to discuss their work, and agreed on a common Turkish version.

Stage 3- Two different back-translations were performed by English, by two independent English native spoken non-medical translators.

Stage 4- An expert committee consisting of a methodologist-biostatistician, four oral health professionals (dental public health, prosthetic dentistry, oral surgery), one linguist and four translators evaluated all reported data. They developed the “pre-final version” of the LORQv3, considering semantic, idiomatic, experiential, and conceptual equivalence. In addition, the face and content validity of the scale were examined by the expert panel in order to assess the clarity of the item wording. At this stage, we contacted the developers of the LORQv3 to avoid misunderstanding and to use correct terminology regarding the term ‘implant retained teeth’. We changed the term ‘implant retained teeth’ to ‘implant retained crown, bridge or denture’ to increase conceptual and semantic equivalence in all related items. To facilitate comprehension, Item 9 ‘Did you have problems drooling?’ was modified to ‘Did the saliva dribble out of the edge of your mouth? and Item 11, ‘Were you upset by your facial appearance?’ was modified to ‘Were you concerned by the appearance of your face?’. To increase conceptual equivalence, Item 15, ‘Did your chewing ability affect your social life?’ was modified to ‘Did you avoid spending time with others in social activities because of your chewing problems?’ and Item 16 ‘Did your chewing ability influence your choice of foods?’ was modified to ‘Did you have a preference for some foods because of your chewing problems?’. The term ‘ulceration’ was translated as ‘any soreness or injury to the gum’ to increase comprehension (Items 27 and 35).

Stage 5- This version was tested on a convenience sample of 12 patients with head and neck cancer to guarantee sensitivity to local culture and selection of the appropriate wording.

Stage 6- Final version of the LORQv3 was sent to the original developer of the LORQv3, for comparison and approval.

### Psychometric validation

#### Subjects

The Turkish version of the LORQv3 was tested in a sample of 46 head and neck cancer patients who underwent oral rehabilitation at the Prosthodontics Clinic of our faculty between January 2008 and December 2010. The sample size was calculated based on the test-retest reliability, which was estimated using weighted kappa. The sample size required that weighted kappa be estimated to be 32, using Cicchetti's Formula: n = 2 k^2^, where *k* is the number of response categories [20]. In order to allow a 20% drop-out between testing and re-testing, at least 38 participants had to be invited.

Participants were retrospectively identified from the patient record information database. Eligibility criteria for participants were: (1) aged 18 years or older; (2) to have received prosthetic rehabilitation with definite obturator prosthesis or conventional prosthesis following cancer treatment; (3) to have been wearing a obturator prosthesis or conventional prosthesis for at least six months; (4) to be disease free at the time of the questionnaire; (5) not to have undergone mandibulectomy nor glossectomy; and (6) to be able to read Turkish. Patients who received postoperative radiotherapy within six months before the HRQOL assessment were excluded.

Sixty-three cancer patients who met these criteria were identified and contacted via telephone by a research assistant. At this initial contact, the research assistant explained the study to the patients and asked them to participate in a research study. Four of these persons had died, five were living too far away, three had continuing or recurrent disease, and five did not want to participate in this study and therefore were not included.

### Procedure

This study was approved by the Ethics Committee of the Faculty of Medicine, the University of Istanbul (Approval number: 2011/502-494), and the patients gave their written consent. Patients who agreed to participate in the study and to visit the clinic twice were invited to our clinic for interviews and clinical examinations. At the first appointment, the charts of enrolled patients were reviewed, and demographic, tumor, and treatment data were collected. The patients were examined by a prosthodontist (A.B.) to assess the size of maxillectomy defect and the status of the dentition. Following examination, questionnaires were administered by a single research assistant (M.O.K.) in the waiting room. For test-retest reliability, all patients filled the LORQv3 twice at 7-14 days intervals in the same waiting room. No treatment was given to the study participants during this two-week period.

### Questionnaire

The questionnaire consisted of the LORQv3, the University of Washington Quality of Life questionnaire version 4 (UW-QOL v4), and a socio-demographic section.

The LORQv3 was developed by Pace-Balzan et al. [15] and consists of 40 items divided into two primary sections. The first 17 items assess issues related to oral function, orofacial appearance and social interaction. The remaining items deal with prostheses and patient denture/prosthetic satisfaction. Items refer to problems or symptoms experienced during the previous week and are rated on a 1–4 Likert scale ranging from never (1) to always (4).

The UW-QOL v4 is a valid and reliable tool specifically designed to assess the quality of life of patients with head and neck malignancies [21]. The UW-QOL v4 consist of 12 disease-specific items divided into two subscales: physical function (chewing, swallowing, speech, taste, saliva, and appearance) and social-emotional function (anxiety, mood, pain, activity, recreation, and shoulder function). Each item is scored from 0 (worst HRQOL) to 100 (best HRQOL). The validity and reliability of the Turkish version of the UW-QOL v4 was tested by Şenkal et al. [22].

### Statistical analysis

Descriptive data included mean values and standard deviation for continuous variables and proportions for categorical variables. Face and content validity of the questionnaire were examined by the expert panel prior to the validation. Reliability was assessed in two ways: internal consistency reliability and test–retest reliability. Internal consistency was evaluated with Cronbach's alpha coefficient. Test- retest reliability, with a 7–14-day interval between test and retest, was measured by the kappa coefficient weighted by applying standard weights according to the number of categories in error. Internal consistency is considered good if Cronbach’s alpha approximates to 0.70 but does not exceed 0.90, which implies the presence of redundant items [23]. Kappa values between 0.00 and 0.20 are considered poor; those between 0.21 and 0.40, fair; those between 0.41 and 0.60, moderate; those between 0.61 to 0.80, good; and those between 0.81 and 1.00, very good [24].

Criterion validity was assessed by the following hypotheses: (1) Significant negative correlations (Spearman’s rank order correlation coefficient) would be found between items of the LORQv3 and the UW-QOL v4 that assess the related function. (2) Items of the LORQv3, assessing the psychosocial impact of prostheses and the participant’s denture satisfaction would be more correlated with the items in the physical subscale of the UW-QOL v4 than the terms in the social–emotional function subscale of the UW-QOL v4. Interpretation of correlation coefficients was as follows: r ≤0.49: weak relationship; 0.50 ≤ r ≤0.74: moderate relationship; and r ≥0.75: strong relationship [25].

Construct validity was evaluated by comparing item scores of the LORQv3 among patient groups who received different oral rehabilitations. It is known that postoperative radiation therapy and the extent of therapy are most important variables affecting HRQOL in maxillectomy patients with prosthetic obturation [5, 6, 8, 9]. Oral cancer survivors had more problems regarding social eating, social contact, and opening the mouth than nasopharyngeal cancer survivors [26]. Thus, patients were classified into three groups: 15 patients rehabilitated with maxillary obturator prostheses after maxillectomy; 23 patients rehabilitated with maxillary obturator prostheses who were treated with surgery plus postoperative radiotherapy, with or without chemotherapy; 8 nasopharyngeal cancer patients without maxillary defects wearing a conventional upper denture, who served as a control group. We hypothesized that: (1) Maxillary obturator patients would report higher scores on the items related to facial appearence, speaking, and dissatisfaction with the upper denture than nasopharynx cancer patients wearing conventional upper dentures; (2) Maxillary obturator patients treated with surgery plus radiotherapy with or without chemotherapy would be more likely to report functional and emotional problems with their upper prosthesis than those who had been treated with surgery alone and who had been diagnosed with nasopharyngeal cancer.

In order to compare the socio-demographic and clinical characteristics of patient groups, continuous data were analyzed by means of analysis of variance (one-way ANOVA), Kruskal-Wallis, Mann- Whitney U and independent- sample *t*-tests. The continuous variables were tested for normal distribution by the Shapiro-Wilk test. Categorical variables were examined using the chi-square test. Fisher’s exact test was used for variables with expected counts <5. To verify differences on LORQv3 scores between groups, we used the non-parametric Kruskal-Wallis test with Dunn's multiple comparison post test. Statistical analyses were performed with SPSS Statistics version 19.0.0 (IBM Corporation, Somers, NY, USA). Linear weighted kappa was calculated according to the procedure given on the website: http://vassarstats.net/kappa.html.

## Results

### Study sample description

The socio-demographic and clinical characteristics of participant groups are shown in Table 1. Of the 46 patients with a mean age of 52.76 ± 12.89 years (range 18-77 years), 8 (17%) had cancers of the nasopharynx and 38 (83%) had cancers of the maxillary sinus. Men made up 63% (n = 29) of the participants; 83% (n = 38) were married; 33% (n = 15) were employed; and 61% (n = 38) had formal school education equal to or less than 8 years. Of the 38 patients with maxillary sinus cancer, 15 (39%) had surgery alone, and 7 (18%) had undergone a facial approach. According to the Brown et al. [27] classification, maxillectomy defects were Class IIb or smaller in 45% (n = 17) and larger than Class IIb in 55% (n = 21) of patients. Tumor classification, according to the American Joint Committee on Cancer (AJCC) staging system, was T1 in 3 patients, T2 in 14 patients, T3 in 16 patients, and T4 in 5 patients. The N classification was NO in 23 patients, N1 in 12 patients, N2a in 2 patient, N2b in 1 patients (data not shown).

**Table 1:** **Socio-demographic and clinical characteristics of study participants and comparisons among the three groups**

	Maxillary sinus cancer rehabilitated with maxillary obturator following surgery alone (A)	Maxillary sinus cancer rehabilitated with maxillary obturator following surgery plus radiotherapy with/without chemotherapy (B)	Nasopharyngeal cancer rehabilitated with conventional prosthesis following radiotherapy with/without chemotherapy (C)	***P***-value
	n = 15	n = 23	n = 8	
**Gender** ^**a**^ **(n, %)**				
Male	11 (73.3)	12 (52.2)	6 (75)	0.311
Female	4 (26.7)	11 (47.8)	2 (25)	
**Marital status** ^**a**^ **(n, %)**				
Single, divorced, or widowed	3 (20)	4 (17.4)	1 (12.5)	0.903
Married	12 (80)	19 (82.6)	7 (87.5)	
**Employment Status** ^**a**^ **(n, %)**				
Employed full-time/part-time	5 (33.3)	9 (39.1)	1 (12.5)	0.383
Unemployed	10 (66.7)	14 (60.9)	7 (87.5)	
**Age** ^**b**^ **(years), Mean (SD)**	57.53 (15.04)	49.21 (11.06)	52.76 (12.89)	0.145
**Monthly Family Income** ^**c**^ **, TRY, Mean (SD)**	1693 (1348.39)	1583.91 (1116.93)	923.75 (281.21)	0.226
**Educational level** ^**a**^ **(n, %)**				
≤ 8 years of schooling	9 (60)	14 (60.9)	5 (62.5)	0.993
> 8 years of schooling	6 (40)	9 (39.1)	3 (37.5)	
**Type of surgery** ^**a**^				
Transoral	13 (86.7)	18 (78.3)	-	0.514
Transfacial	2 (13.3)	5 (21.7)		
**Defect size** ^**a**^				
Class ≤2 b	8 (53.3)	9 (39.1)	-	0.389
Class >2 b	7 (46.7)	14 (60.9)		

SD, standard deviation; TRY, Turkish Lira; ^a^Statistical evaluation by the chi-square test; ^b^Statistical evaluation by the one-way ANOVA test; ^c^Statistical evaluation by the Kruskal-Wallis test.

According to the AJCC classification staging system for nasopharyngeal carcinoma, three patients (38%) had T1, 3 (38%) had T2, one (12%) had T3 and one (12%) had T4 tumors. Two patients (25%) had N0, four (50%) had N1, and two (25%) had N2. Three patients (37%) were treated with radiotherapy alone and five patients (63%) were treated with radiotherapy plus chemotherapy (data not shown).

There were no statistically significant differences among all patient groups with regards to socio-demographic characteristics. Additionally, no significant differences were found in demographic and clinical characteristics between the two groups of patients rehabilitated with a maxillary obturator.

### Psychometric properties of the LORQv3

Cronbach’s alpha for internal consistency of items was 0.82 for the first 17 LORQv3 items. For Items 20–23, Cronbach’s alpha was 0.72, for Items 26–31 alpha was 0.77 and for Items 34–39 alpha was 0.71.

The first 17 items of the LORQv3 assessed issues relating to oral function, orofacial appearance and social interaction and were applicable to all patients (Table 2). All maxillary sinus cancer patients were rehabilitated with conventional obturator prostheses. There were relatively fewer nasopharyngeal cancer patients with natural teeth in their upper jaw than patients with maxillary sinus cancer. More than half of the patients in each group still had natural teeth in either the upper or the lower jaw. Implant retained crowns, bridges or dentures in both jaws were not found in the patient groups (Table 3).

**Table 2:** **The differences for the first 17 LORQv3 items assessing issues related to oral function, orofacial appearance and social interaction among patient groups**

		Maxillary sinus cancer rehabilitated with maxillary obturator following surgery alone (A)		Maxillary sinus cancer rehabilitated with maxillary obturator following surgery plus radiotherapy with/without chemotherapy (B)		Nasopharyngeal cancer rehabilitated with conventional prosthesis following radiotherapy with/without chemotherapy (C)	
		n = 15		n = 23		n = 8	
		Mean	SD	Mean	SD	Mean	SD
1	Did you experience difficulty chewing?	2.26	1.16	2.69	0.97	2.75	1.28
2	Did you have pain when you chew?	2.00	1.33	1.95	0.70	1.75	1.16
3	Did you experience difficulty swallowing solids?*	1.60	0.91	2.39	1.03	2.62	1.18
4	Did you experience difficulty swallowing drinks?	1.86	0.99	1.65	0.93	1.87	1.12
5	Did food particles collect under your tongue?	1.33	0.82	1.47	0.73	1.75	0.70
6	Did food particles stick to your palate?	2.20	0.94	2.22	0.85	2.12	1.12
7	Did food particles stick inside your cheeks?	1.73	0.96	1.86	0.75	2.00	1.19
8	Did you have mouth dryness?**	1.80	0.67	2.86	1.05	3.00	1.30
9	Did the saliva dribble out of the edge of your mouth?	1.46	0.91	1.91	0.84	1.37	0.74
10	Did you have problems when speaking?*	2.20	0.94	2.47	0.94	1.50	0.53
11	Were you concerned by the appearance of your face?**	2.06	1.16	2.21	0.95	1.12	0.35
12	Were you concerned by the appearance of your mouth?	2.00	1.06	2.04	1.06	1.50	0.75
13	Were you upset by the appearance of your lips?	1.60	1.12	1.95	0.97	1.37	0.51
14	Were you upset by the appearance of your teeth?	1.73	0.96	1.74	1.09	1.37	0.74
15	Did you avoid spending time with others in social activities because of your chewing problems?	1.93	0.96	1.78	1.08	2.00	1.06
16	Did you have a preference for some foods because of your chewing problems?	2.00	1.06	2.30	1.01	2.75	1.28
17	Did you experience difficulty with opening your mouth?***	1.20	0.41	2.60	1.07	3.12	0.83

SD, standard deviation; Kruskal-Wallis test for differences between 3 groups: *P < 0.05, **P < 0.01, ***P < 0.001**.** Post hoc comparisons using Dunn's test revealed following significant differences among groups at p <0.05 significance level: Group A vs Group B, Group A vs Group C for item 3; Group A vs Group B, Group A vs Group C, Group B vs Group C for item 8, Group B vs Group C for item 10; Group A vs Group C, Group B vs Group C for item 11; and Group A vs Group B, Group A vs Group C for item 17.

**Table 3:** **LORQv3 items dealing with presence of natural teeth, dentures and implant retained teeth**

	Maxillary sinus cancer rehabilitated with maxillary obturator following surgery alone (A)		Maxillary sinus cancer rehabilitated with maxillary obturator following surgery plus radiotherapy with/without chemotherapy (B)		Nasopharyngeal cancer rehabilitated with conventional prosthesis following radiotherapy with/without chemotherapy (C)	
	n = 15		n = 23		n = 8	
	%	n	%	n	%	n
18. Do you have any natural teeth in the UPPER jaw?	67	10/15	52	12/23	63	5/8
19. Do you have any natural teeth in the LOWER jaw?	80	12/15	87	20/23	50	4/8
Natural teeth in either upper or lower jaw	53	8/15	52	12/23	50	4/8
24. Do you have an UPPER denture?	100	15/15	100	23/23	100	8/8
25. Do you have UPPER implant retained crown, bridge or denture?	0		0		0	
Upper denture or implant retained teeth	100	15/15	100	23/23	100	8/8
32. Do you have a LOWER denture?	60	9/15	43	10/23	50	4/8
33. Do you have LOWER implant retained crown, bridge or denture?	0		0		0	
Lower denture or implant retained teeth	60	9/15	43	10/23	50	4/8

Regarding construct validity, we found significant differences in some items related to swallowing solid, dry mouth, speaking, facial appearance and mouth opening among patient groups. Maxillary obturator patients who received surgery alone scored better than those with radiotherapy on three items: mouth dryness*,* difficulty with swallowing solids*,* and opening the mouth. Differences between maxillary obturator patients and nasopharyngeal cancer patients were specific to certain items and were consistently worse for the maxillary obturator patients - problems when speaking and concern about facial appearance. Nasopharyngeal cancer patients reported more difficulties with mouth dryness (xerostomia) and opening the mouth (Table 2).

The second part of the LORQv3 assessed the social impact of prostheses and the patient’s denture satisfaction. Maxillary obturator patients who received surgery alone reported less difficulty with opening mouth than patients who had received radiotherapy (Table 4).

**Table 4:** **Differences for items 20 to 23, dealing with prostheses and satisfaction among study groups**

	Maxillary sinus cancer rehabilitated with maxillary obturator following surgery alone (A)		Maxillary sinus cancer rehabilitated with maxillary obturator following surgery plus radiotherapy with/without chemotherapy (B)		Nasopharyngeal cancer rehabilitated with conventional prosthesis following radiotherapy with/without chemotherapy (C)	
	n = 15		n = 23		n = 8	
	Mean	SD	Mean	SD	Mean	SD
If dentures or implant retained crown, bridges or dentures (YES to question 24, 25, 32 or 33)						
20. Were you embarrassed talking with others because of your dentures/implant retained crown, bridges or dentures?						
	1.86	0.83	1.47	0.94	1.62	0.74
21. Did you refuse dinner invitations because of feeling embarrassed about your dentures/implant retained crown, bridges or dentures?						
	1.60	0.63	1.73	1.09	2.00	1.06
22. Did you feel loss of self-confidence because of embarrassment about your dentures/implant retained crown, bridges or dentures?						
	1.46	0.51	1.34	0.48	1.25	0.46
23. Did you have difficulty opening mouth because of your dentures/implant retained crown, bridges or dentures?***						
	1.20	0.41	2.17	0.83	2.25	0.46

SD, standard deviation; Kruskal-Wallis test for differences between 3 groups: ***P < 0.001. Post hoc comparisons using Dunn's test revealed following significant differences among groups at p **<**0.05 significance level: Group A vs Group B, Group A vs Group C for item 23.

For items 26 to 31, referring to maxillary dentures or implant-retained teeth, maxillary obturator patients generally felt more dissatisfied and insecure than nasopharynx cancer patients without any maxillary defects. Compared with nasopharyngeal cancer patients, maxillary obturator patients who received surgery plus radiotherapy felt more concerned about their dentures and reported having soreness or injury to the gums due to their dentures; they were the most likely to find food collecting under their dentures (Table 5).

**Table 5:** **Differences for items 26 to 31, referring to maxillary dentures or implant-retained teeth, among study groups**

	Maxillary sinus cancer rehabilitated with maxillary obturator following surgery alone (A)		Maxillary sinus cancer rehabilitated with maxillary obturator following surgery plus radiotherapy with/without chemotherapy (B)		Nasopharyngeal cancer rehabilitated with conventional prosthesis following radiotherapy with/without chemotherapy (C)	
	n = 15		n = 23		n = 8	
	Mean	SD	Mean	SD	Mean	SD
If upper dentures or implant retained crown, bridges or dentures (YES to question 24 or question 25)						
26. Were you dissatisfied with your upper denture/implant retained crown, bridges or dentures?**	2.66	0.97	2.39	0.94	1.25	0.46
27. Did your upper denture/ implant retained crown, bridges or dentures cause any soreness or injury to the gum?**	2.20	0.86	2.52	0.89	1.37	0.51
28. Did food particles collect under your upper denture/implant retained crown, bridges or dentures?*	2.53	0.74	2.95	0.87	1.87	0.99
29. Did you take out your upper denture/implant retained crown, bridges or dentures for eating?	1.53	0.91	1.34	0.77	1.25	0.46
30. Did you feel anxious with your upper denture/implant retained crown, bridges or dentures?**	2.53	0.99	2.78	0.73	1.62	0.51
31. Were you worried about your upper denture/implant retained crown, bridges or dentures?*	2.26	0.79	2.73	1.05	1.62	0.74

SD, standard deviation; Kruskal-Wallis test for differences between 3 groups: *P < 0.05, **P < 0.01. Post hoc comparisons using Dunn's test revealed following significant differences among groups at p <0.05 significance level: Group A vs Group C, Group B vs Group C for item 26; Group B vs Group C for item 27; Group B vs Group C for item 28; Group A vs Group C, Group B vs Group C for item 30; Group B vs Group C for item 31.

For items 34 to 39, concerning mandibular dentures or implant-retained teeth groups, the groups of patients who received radiotherapy felt more worried about their lower dentures and were the most likely to find food collecting under these and to have soreness or gingival injury than maxillary obturator patients who received surgery alone (Table 6).

**Table 6:** **Differences for items 34 to 39, referring to mandibular dentures or implant-retained teeth among patient groups**

	Maxillary sinus cancer rehabilitated with maxillary obturator following surgery alone (A)		Maxillary sinus cancer rehabilitated with maxillary obturator following surgery plus radiotherapy with/without chemotherapy (B)		Nasopharyngeal cancer rehabilitated with conventional prosthesis following radiotherapy with/without chemotherapy (C)	
	n = 9		n = 10		n = 7	
	Mean	SD	Mean	SD	Mean	SD
If lower dentures or implant retained crown, bridges or dentures (YES to question 32 or question 33)						
34. Were you dissatisfied with your lower denture/implant retained crown, bridges or dentures?	1.88	1.36	1.70	0.48	2.14	1.21
35. Did your lower denture/ implant retained crown, bridges or dentures cause any soreness or injury to the gum?**	1.22	0.44	2.20	0.42	3.00	0.81
36. Did food particles collect under your lower denture/implant retained crown, bridges or dentures?**	1.33	0.50	3.10	0.32	3.00	0.82
37. Did you take out your lower denture/implant retained crown, bridges or dentures for eating?	2.33	0.86	2.40	0.51	2.14	1.21
38. Did you feel unconfident with your lower denture/implant retained crown, bridges or dentures?	1.33	0.50	1.80	0.42	1.71	0.75
39. Were you worried about your lower denture/implant retained crown, bridges or dentures?**	1.77	0.44	3.00	0.82	2.71	0.48

SD, standard deviation; Kruskal-Wallis test for differences between 3 groups: **P < 0.01; Post hoc comparisons using Dunn's test revealed following significant differences among groups at p <0.05 significance level: Group A vs Group B, Group A vs Group C, Group B vs Group C for item 35; Group A vs Group B, Group A vs Group C for item 36; Group A vs Group B, Group A vs Group C for item 39.

All patients participated in the test-retest, with a 10-day median interval (interquartile range: 8 to 12 days). For the 33 LORQ items, kappa ranged from 0.62 to 1.00, which represents 'good' to 'perfect’ agreement and none demonstrated poor or fair agreement. In the first section of the LORQv3, all items showed good to perfect agreement. Of the 16 items in the second section of the LORQv3, only seven had good agreement, with the remaining nine items having “perfect” agreement. For the 33 LORQ items, median kappa was 0.79, interquartile range was 0.71 to 0.85, and range was 0.62 to 1.00. Median kappa for the first 17 items was 0.75 and for the other items, 0.84 (data not shown).

Criterion validity was supported via correlations for some related items between the LORQv3 and the UW-QOL v4 (Table 7). Out of a total of 396 correlations, we found a total of 96 statistically significant negative correlations between the LORQv3 items and the UW-QOL v4 item scores (median –0.39, interquartile range (IQR) –0.44 to –0.34). Consistent with the first hypothesis, moderate to weakly significant correlations were observed for some related domains between the LORQv3 and the UW-QOL v4: The UWQoLv4 appearance item correlated with all LORQv3 items (Items 11,12,13 and 14) concerning appearance (range of r = –0.30 to –0.51; *P* < 0.05 to *P* <0.01); the UWQoLv4 saliva item correlated with some LORQv3 items (Items 3,5,8,16,17,23,31,35, 36 and 39) about the problems associated with xerostomia (range –0.32 to –0.51; *P* <0.05 to *P* <0.01); the UWQoLv4 chewing item correlated with all LORQv3 items related to chewing problems (range -0.43 to -0.60; *P* <0.01); the UWQoLv4 swallowing item correlated with Item 3 about swallowing (r = –0.65; *P* <0.01); the UWQoLv4 pain item correlated with the swallowing Items 3 and 4 of the LORQv3 (r = –0.38; *P* <0.01 and r = –0.44; *P* <0.01, respectively).

**Table 7:** **Correlations between items of the LORQv3 and UW-QOL v4**

LORQv3	Pain	Appearance	Activity	Recreation	Swallowing	Chewing	Speech	Shoulder	Taste	Saliva	Mood	Anxiety
1. … difficulty chewing?	-0.18	-,45**	-,31*	-0.27	-,32*	-,55**	-0.19	-0.15	-,37*	-0.13	-0.21	0.04
2. … pain when you chew?	-0.27	-0.24	-0.02	-0.04	-0.05	-,33*	-0.04	-0.17	-0.18	-0.07	-0.19	-0.01
3. … difficulty swallowing solids?	-,38**	-,37*	-,47**	-,37*	-,65**	-,49**	-,46**	-0.17	-,37*	-,41**	-0.25	-0.13
4. … difficulty swallowing drinks?	-,44**	0.09	-0.09	-0.11	-0.09	0.09	0.02	-0.18	-0.06	0.00	-0.21	-0.21
5. …particles collect under your tongue?	-0.07	-0.04	-0.01	0.10	-0.11	-,38**	0.06	-0.26	-0.05	-,48**	-0.02	0.12
6. …particles stick to your palate?	0.03	0.03	0.10	-0.08	0.09	0.08	,33*	-0.12	-0.02	-0.08	-0.22	0.04
7. …particles stick inside your cheeks?	-0.01	0.08	0.14	-,37*	-0.09	0.02	0.18	0.00	-0.05	0.07	0.12	0.10
8. …have mouth dryness?	-0.06	-0.19	-,41**	-0.23	-0.25	-0.26	-0.23	0.12	-0.22	-,48**	-0.08	-0.03
9. ....saliva dribble out of the edge of your mouth	-0.28	-,41**	-0.23	-0.19	-0.16	-0.26	-,40**	0.02	-0.26	-0.26	-0.11	-,38**
10. . problems when speaking?	-0.18	-,39**	-0.23	-,36*	-0.03	-0.24	-0.26	-0.01	-0.05	-0.16	-0.19	-,31*
11. . concerned by the appearance of your face?	0.04	-,49**	-0.03	-0.24	0.21	-0.09	-0.10	0.28	0.03	0.13	-0.07	-0.06
12. . concerned by the appearance of your mouth?	-0.12	-,51**	-0.21	-0.27	-0.07	-0.25	-0.22	-0.04	-0.23	0.07	-,37*	-0.09
13. .upset by the appearance of your lips?	-0.28	-,44**	-0.28	-0.24	-0.12	-,30*	-0.19	0.05	-0.23	0.01	-0.18	-0.08
14. .upset by the appearance of your teeth?	-0.21	-,30*	-0.26	-0.23	-0.02	-0.20	-0.15	-0.03	-0.14	-0.03	-0.21	-0.03
15. . spending time with others in social activities..?	-0.17	-,43**	-0.25	-,39**	-0.16	-,43**	-,37*	-0.16	-,34*	-0.19	-,40**	0.01
16. . preference for some foods because of chewing …?	-0.18	-,39*	-0.24	-,30*	-0.27	-,60**	-,41**	-0.28	-,48**	-,35*	-,30*	0.05
17. . experience difficulty with opening your mouth?	-0.07	-0.27	-0.29	-0.19	-0.21	-0.17	-0.19	0.15	-0.20	-,32*	-0.15	-0.03
20. . embarrassed talking with others… of your dentures/..?	-,41**	-,40**	-,30*	-,36*	-0.05	-0.13	-,33*	-0.07	-0.13	-0.08	-0.27	-0.14
21. . refuse dinner invitations……..about your dentures?	-0.26	-,35*	-,39**	-,36*	-,36*	-0.23	-,36*	-0.12	-,37*	-0.29	-0.21	0.08
22. .loss of self confidence …….about your dentures/…?	-0.06	-,44**	-0.08	-,32*	0.18	-0.02	-0.08	0.16	0.02	0.06	-0.12	-0.09
23. .difficulty opening mouth because of you dentures/imp…?	-0.21	-,29*	-0.19	-0.10	-0.16	-0.21	-0.25	-0.01	-0.21	-,49**	0.09	0.02
26. . dissatisfied with your upper denture/…?	-,41**	-,40**	-,30*	-,36*	-0.05	-0.13	-,33*	-0.07	-0.13	-0.08	-0.27	-0.14
27. . cause any soreness or injury to the gum?	-0.20	-0.20	-0.25	-0.26	-0.27	-0.21	-0.21	-0.01	-,34*	-0.13	-0.08	-0.07
28. . particles collect under your upper denture/…?	-,32*	-,35*	-,32*	-0.28	-,42**	-,30*	-,38**	-0.01	-,39**	-0.27	-0.15	-0.04
29. . take out your upper denture/implant…?	-0.16	0.00	-0.20	-0.05	-0.22	-0.04	-0.25	-,31*	-0.06	-0.11	-0.08	0.08
30. . anxious with your upper denture/implant..?	-0.22	-0.26	-0.25	-,32*	-,30*	-0.11	-0.27	-0.01	-0.26	-0.13	-0.07	-0.02
31. . worried about your upper denture/implant…?	-0.11	-0.14	-0.12	-0.06	-0.12	-0.15	-0.15	0.01	-0.09	-,45**	0.15	0.02
34. . dissatisfied with your lower denture/ implant..?	-,42*	-0.31	-0.04	-0.03	-0.16	-0.18	-0.35	-,40*	-0.17	0.07	-0.24	-0.19
35. . any soreness or injury to the gum…?	-0.21	-0.25	-0.16	-0.20	-0.32	-,43*	-0.37	-0.11	-,45*	-,45*	-0.03	0.26
36. . particles collect under your lower denture/…?	-0.16	-0.15	-0.07	-0.03	-,43*	-,52**	-0.09	-0.05	-0.19	-,51**	0.04	0.21
37. . take out your lower denture/implant…?	0.18	-,44*	-0.08	-0.27	-0.21	0.04	-0.18	0.04	-0.01	0.01	-0.35	-0.25
38. . unconfident with your lower denture….?	-,40*	-,42*	-0.23	-0.31	-,39*	-,46*	-,44*	-0.29	-0.34	-0.28	-,42*	-0.17
39. . worried about your lower denture/implant…..?	-0.18	-0.28	-0.04	-0.16	-0.29	-0.30	-0.24	-0.05	-0.22	-,40*	-0.10	0.24

**Correlation is significant at the 0.01 level (2-tailed); *Correlation is significant at the 0.05 level (2-tailed).

Consistent with the second hypothesis, we found the following correlations: LORQv3 items, assessing psychosocial impact of dentures and patient’s satisfaction, were more correlated with the items in the physical function subscale of the UWQoLv4 than the items in the social-emotional subscale.

## Discussion

HRQOL has become an important outcome measure for assessing and monitoring the impacts of oral rehabilitation on the subjective well-being of patients with head and neck cancer [5–11]. However, a small number of studies have assessed the HRQOL using oral health specific and generic measures in Turkish patients with head and neck cancer who had undergone oral rehabilitation [28, 29], whereas only one study has assessed HRQOL using a targeted measure in patients with implant-retained maxillofacial prostheses [30].

To the best of our knowledge, this is the first study to evaluate the HRQOL using a head and neck function specific measure in Turkish patients with head and neck cancer.

As with many such instruments, this scale was developed in English and requires translation and validation in Turkish if it is to be used in that language. In the present study, the original LORQv3 was translated into Turkish, following the recommendations of Beaton et al. [19] and resulted in a back-translated version that was very similar to the original, although word modifications were made to take into account cultural differences.

In contrast to previous studies of the LORQv3 [15, 16], this study sample consisted of only head and neck cancer patients who had undergone prosthodontic treatment at the Prosthodontics Clinic of our faculty, because patients with head and neck cancer have very specific needs that are beyond the needs of most other patients diagnosed with other types of cancer.

The reliability analysis showed that all sections of the LORQv3 showed satisfactory internal consistency, with Cronbach’s alpha between 0.71 to 0.82. Our findings are consistent with those of previous studies [15, 16]. In the test-retest reliability, kappa statistics for the 33 LORQ items ranged from 0.62 to 1.00, indicating good reproducibility.

Construct validity was supported by comparing item scores of the LORQv3 among patient groups consisting of patients with maxillectomy and nasopharyngeal cancer without any maxillary defects. Patients who had undergone mandibulectomy or glossectomy were not included in this study, because the factors affecting HRQOL in these patients were different from those in the maxillectomy patients [31].

In patients with maxillectomy, obturators are important not only in rehabilitation and aesthetics, but also in patient resocialization [5, 6, 8, 9]. Consistent with previous studies, we found that maxillectomy patients who received adjuvant radiation experienced dry mouth [5], difficulties in mouth opening [8] and swallowing [8, 10].

Supporting construct validity of LORQv3, we found significant differences on the items regarding with being concerned about appearance and the problems when speaking between maxillary obturator patients and nasopharyngeal cancer patients. Consistent with previous studies, maxillary obturator patients who received surgery alone scored better than those who had been treated with radiotherapy on three items: mouth dryness [5] *,* difficulty with swallowing solids [8, 10, 11] and opening the mouth [8]. Maxillary obturator patients generally felt more dissatisfied and anxious than nasopharyngeal cancer patients. A previous study [26] showed that oral cancer patients experienced more social problems than nasopharyngeal cancer patients. Compared with nasopharyngeal cancer patients, maxillary obturator patients who received surgery plus radiotherapy felt more worried about their dentures and reported having pain or gingival injury due to the dentures and were the most likely to find food collecting under the dentures. These findings may be explained by the fact that functional challenges in patients with maxillectomy that limit the ability to speak and eat are often apparent and lead to social isolation, loss of employment, and decreased quality of life [7, 9, 10]. In these patients, adjuvant radiotherapy may result in greater self-reported oral and swallowing problems [1, 5, 7, 8].

We found no new items related to oral rehabilitation because no participant provided free-text comments, supporting content validity of the LORQv3.

Supporting its criterion validity, significant negative correlations were observed for some related items between the LORQv3 and the UW-QOL v4, which are in line with the results of a previous study [15]. Items of the LORQv3, assessing the psychosocial function and patient satisfaction correlated better with the items on the physical subscale of the UW-QOL v4 than with its items on the social–emotional function subscale. This finding is not surprising, because the study sample included predominantly maxillectomy patients, rehabilitated with obturator. Previous studies showed that inadequate orofacial functions, essential for social well being, that are affected by obturator stability and retention are the most important problems faced by maxillectomy patients rehabilitated with obturator [5, 9, 10]. These findings support the concept of using a head and neck specific questionnaire as an indicator of oral function.

There are some limitations to this study. This study involved a small cohort of patients comprised of patients with maxillary sinus and nasopharyngeal cancer. Future studies are needed to evaluate psychometric properties of the LORQv3 in both patients diagnosed with other types of head and neck cancer and non-cancer patients. The cross-sectional design did not allow causation or changes over time in patients’ HRQOL to be studied. Future clinical and longitudinal studies using the LORQv3 may provide valuable information for the maxillofacial prosthetic team in assessing self-perceived change of HRQOL in these patients.

Despite these limitations, this study has strengths. Firstly, a sample size was calculated based on the test–retest reliability in accordance with a recent systematic review of validation studies of cancer patients under palliative care [32]. It is known that the ability of a measure to be responsive to change depends upon test-retest reliability and better reproducibility, suggesting more precise single measurements, which is a requirement for better tracking of changes in measurements in clinical practice settings [32, 33]. Secondly, patients who had continuing or recurrent disease were excluded from the study, because retested patients must be in a stable condition with respect to the construct to be measured by the HRQOL measures [2, 32]. Lastly, the appropriate time interval of two weeks was chosen because it depends on the construct to be measured and the target population [14, 16, 32].

## Conclusion

Within the limitations of this study, the Turkish version of LORQv3 appears to be a valid and reliable tool for assessing the impact of oral rehabilitation on the HRQOL of patients with head and neck cancer; it can also be used in clinical investigations and routine clinical practice.

## Abbreviations

HRQOL: Health-related quality of life
LORQv3: The Liverpool Oral Rehabilitation Questionnaire version3
T1: Translator 1
T2: Translator 2
UW-QOL v4: The University of Washington Quality of Life questionnaire, version 4
AJCC: The American Joint Committee on Cancer.
